# The Neuroimmune Mechanisms Linking Smoking and Infection to Atherosclerosis: Chronic vs. Surge

**DOI:** 10.1007/s12012-026-10155-2

**Published:** 2026-07-11

**Authors:** Paulo Roberto Benchimol-Barbosa

**Affiliations:** Department of Cardiology, Clinical Staff Services, Hospital Universitário Pedro Ernesto, Rio de Janeiro State University, Boulevard Vinte e Oito de Setembro, 77/Ground floor, Board of Directors Suite, Vila Isabel, Rio de Janeiro, RJ 20551-030 Brazil

**Keywords:** Smoking, Systemic inflammation, Atherogenesis, Cardiovascular events, Infection

## Abstract

The pathogenesis of atherosclerosis has progressively shifted from a lipid-centric model to one that recognizes inflammation as the central driver. Smoking is a well-established chronic risk factor, whereas acute infections are increasingly recognized as transient triggers of acute cardiovascular events. This review synthesizes the distinct yet overlapping mechanisms by which chronic smoking and acute infections contribute to atherogenesis and plaque destabilization within an integrated neuroimmune framework. This narrative review was developed using AI-assisted literature search tools (ChatGPT, OpenAI; Claude, Anthropic) to identify relevant publications from 1990 to 2025. All references were independently verified using PubMed and original source links. Selection prioritized mechanistic studies addressing inflammatory pathways, endothelial dysfunction, and immune modulation. Smoking promotes atherogenesis through sustained endothelial injury, lipoprotein oxidation, chronic sympathetic activation, and hematopoietic reprogramming, favoring proinflammatory monocyte mobilization. Infections elicit acute cytokine surges, sympathetic-mediated macrophage activation, and prothrombotic cascades that destabilize preexisting plaques. Smoking contributes to plaque initiation and growth, whereas infections primarily act as event triggers. Atherosclerosis emerges from chronic and episodic inflammatory processes with distinct temporal profiles. Smoking is a continuous driver of plaque formation, whereas acute infections promote plaque destabilization. Future research should examine their interplay, particularly in smokers exposed to recurrent or low-grade infectious states.

**Graphical Abstract:**

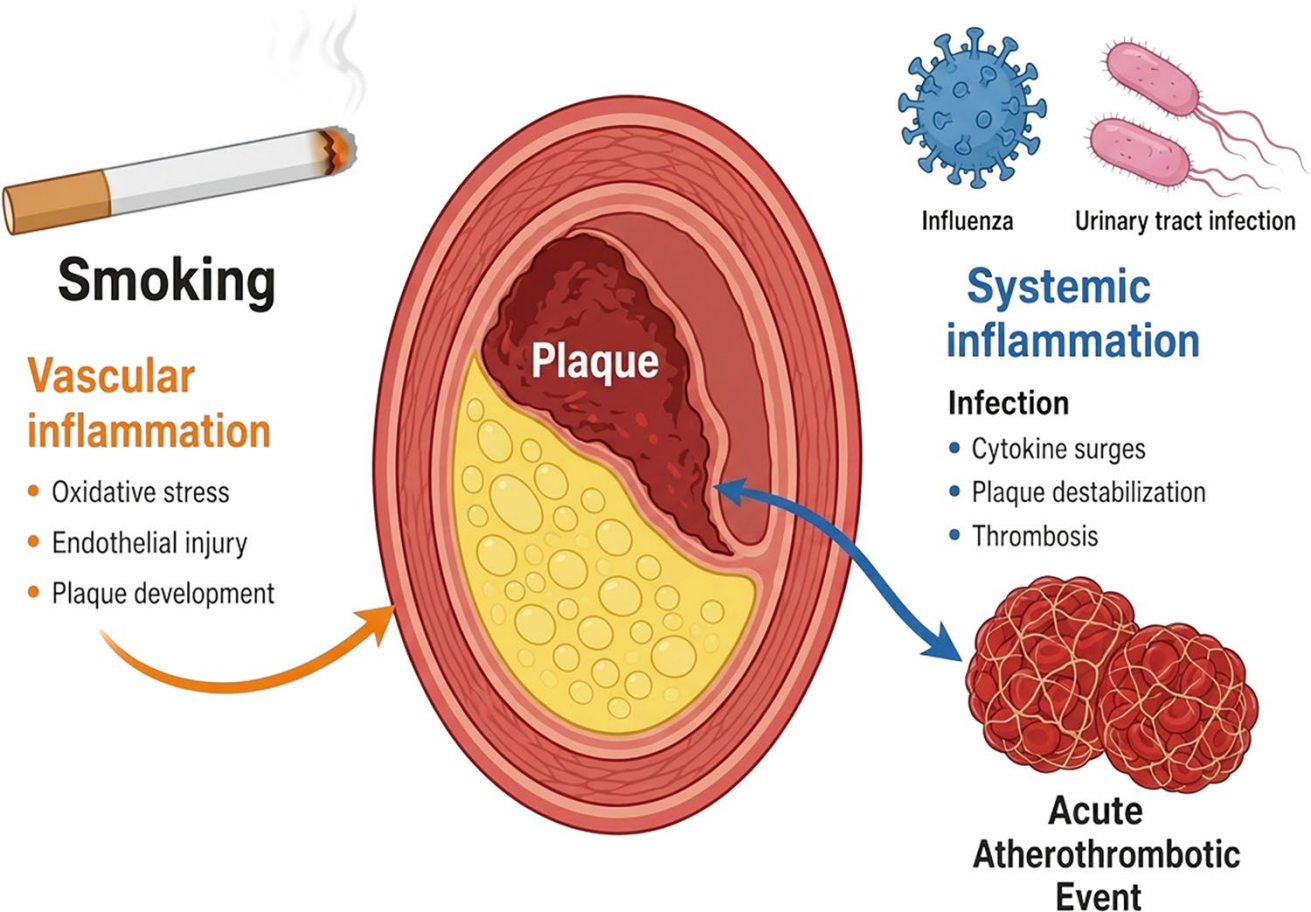

## Introduction

The conceptual comprehension of atherogenesis has evolved considerably over the past two centuries. In the 19th century, Virchow advanced the theory that inflammation and immune cell infiltration are pivotal in vascular injury and lesion formation [1]. In contrast, Carl von Rokitansky proposed that atherosclerosis primarily results from passive deposition of lipids and fibrin within the arterial wall [1]. These contrasting models of active versus passive pathogenesis have coexisted for decades, shaping early clinical reasoning regarding vascular diseases. It was not until the latter half of the 20th century, with advances in vascular biology and immunology, that a consensus emerged favoring an immunoinflammatory paradigm of atherogenesis [2].

At the beginning of the 20th century, cardiovascular disease (CVD) was not the leading cause of death worldwide. Infectious diseases, such as tuberculosis, influenza, and pneumonia, account for most fatalities, particularly in younger populations [3, 4]. However, this landscape began to shift dramatically with urbanization, industrialization, and increased life expectancy, collectively reshaping global health priorities [5, 6]. By the 1930 s and the 1940 s, autopsy series and hospital records increasingly reported coronary thrombosis and myocardial infarction in middle-aged adults, particularly in Western countries [7]. Clinicians have begun to recognize the growing epidemic of cardiovascular morbidity, although they still lack a clear understanding of its risk factors, natural history, and pathophysiological basis [8].

This epidemiological transition spurred the establishment of the Framingham Heart Study in 1948, one of the first prospective cohort studies to focus on cardiovascular risk. Over the ensuing decades, its findings revolutionized preventive cardiology by identifying smoking, hypercholesterolemia, hypertension, and diabetes as potentially modifiable contributors to myocardial infarction and stroke [9].

Although age-standardized cardiovascular mortality has declined in many high-income countries, the absolute global burden of CVD has surged to unprecedented levels. From a relatively infrequent cause of death in the early 1900 s, CVD now accounts for over 20 million deaths annually worldwide, largely due to population aging, dietary transitions, sedentary lifestyles, and the global spread of cardiometabolic risk factors [10].

As cardiovascular research has evolved from descriptive epidemiology to mechanistic investigation, attention has turned to understanding how specific exposures exert pathogenic effects. Cigarette smoking has emerged as a leading modifiable behavioral risk factor and a potent, sustained vascular inflammatory trigger [11]. Mechanistic studies have shown that smoking impairs endothelial function, promotes the oxidative modification of lipoproteins, and sustains low-grade vascular inflammation, which are hallmarks of early and progressive atherogenesis. Thus, smoking exemplifies a chronic, vascular-targeted inflammatory stimulus that can initiate and propagate plaque formation.

In contrast, recent studies have highlighted non-smoking sources of systemic inflammation, such as respiratory or urinary tract infections and autoimmune flares, may act as episodic, yet significant, amplifiers of cardiovascular risk. These transient inflammatory episodes can destabilize pre-existing plaques, trigger prothrombotic cascades, and precipitate acute coronary events. Epidemiological data suggest that the risk of myocardial infarction increases notably in the days to weeks following such events [12, 13].

Together, these insights support a dual inflammatory model of atherosclerosis, in which both chronic exposure (particularly smoking) and acute inflammatory surges (such as infections) act through distinct but converging pathways that drive plaque formation, destabilization, and clinical expression of the disease. Although the dual inflammatory model of atherosclerosis is well-established, a critical gap persists in the comprehensive integration of the distinct and convergent neuroimmune mechanisms by which chronic smoking and acute infections differentially drive atherogenesis. Further exploration of this nexus could enhance our understanding of disease progression and contribute to the development of targeted therapeutic strategies.

To address this gap, this review aims to advance our understanding of disease progression by providing a detailed and integrated neuroimmune framework that explains the differential but convergent roles of chronic smoking (as a sustained vascular insult) and acute infections (as episodic systemic triggers), thereby advising the development of novel diagnostic and therapeutic strategies. The atherogenic mechanisms associated with dyslipidemia, including oxidative modification and subendothelial retention of low-density lipoproteins, scavenger receptor-mediated uptake, and foam cell development, as well as those related to diabetes mellitus, such as advanced glycation end-product accumulation and metabolic inflammation, hypertension, including endothelial dysfunction and enhanced transendothelial migration of inflammatory cells, and obesity, through adipokine dysregulation, chronic low-grade inflammation, and perivascular adipose tissue dysfunction, represent equally important but mechanistically distinct pathways. In-depth reviews of these mechanisms may be found elsewhere [14–16]. In parallel with these classical pathways, direct microbial colonization of the vessel wall and smoking-related microbial dysbiosis have been proposed as complementary contributors to atherogenesis and are briefly addressed in Sect. "Pathogen-Driven Atherogenesis and Smoking-Induced Microbial Dysbiosis".

## Methods

This narrative review synthesizes evidence on the contribution of smoking to chronic vascular inflammation and atherosclerotic plaque formation and of infection-related systemic inflammation to atherogenesis, plaque destabilization, and acute cardiovascular events.

Thematic domains were defined as follows: (1) chronic inflammation associated with smoking; (2) acute systemic inflammation related to infection; and (3) cardiovascular outcomes involving atherogenesis, plaque destabilization, or clinical events.

Literature exploration was conducted using AI-assisted tools (ChatGPT, OpenAI; Claude, Anthropic) to search for relevant publications from inception to 2025, emphasizing those from 1990. Selected educational and governmental sites with relevant historical and epidemiological information were also assessed. This approach was chosen for its efficiency in identifying thematically relevant studies across a broad mechanistic landscape and facilitating a conceptual synthesis. Recognizing that AI tools may generate inaccurate references or favor widely cited studies over less prominent but relevant work, all AI-suggested references were independently validated by the author using PubMed database. This validation involved cross-referencing titles, authors, and publication details, as well as a brief review of abstracts to confirm direct relevance and assess the methodological quality of the source study prior to inclusion. A formal risk of bias assessment using systematic review tools was not performed, consistent with the scope of a narrative review.

Final inclusion was based on mechanistic relevance to inflammatory pathways, endothelial dysfunction, oxidative stress, cytokine signaling, or vascular immune responses, and historical and epidemiological information in alignment with the review’s conceptual framework. Priority was given to mechanistic studies, translational research, and peer-reviewed articles published in English. Landmark studies and websites were included to contextualize the evolution of current understanding.

## Smoking-Induced Atherogenesis: Mechanistic Evolution and Current Understanding

The recognition of smoking as a cardiovascular risk factor dates back to mid-20th-century cohort studies, such as the Framingham Heart Study^9^. However, detailed mechanistic insights have recently emerged with advances in molecular biology in the 1980 s and the 1990s. A convergence of findings from epidemiology, animal models, histopathology, and biomarker analyses has confirmed that cigarette smoking promotes atherogenesis via several interrelated and biologically plausible pathways [11].

### Endothelial Dysfunction and Immune Activation

**Fig. 1:**
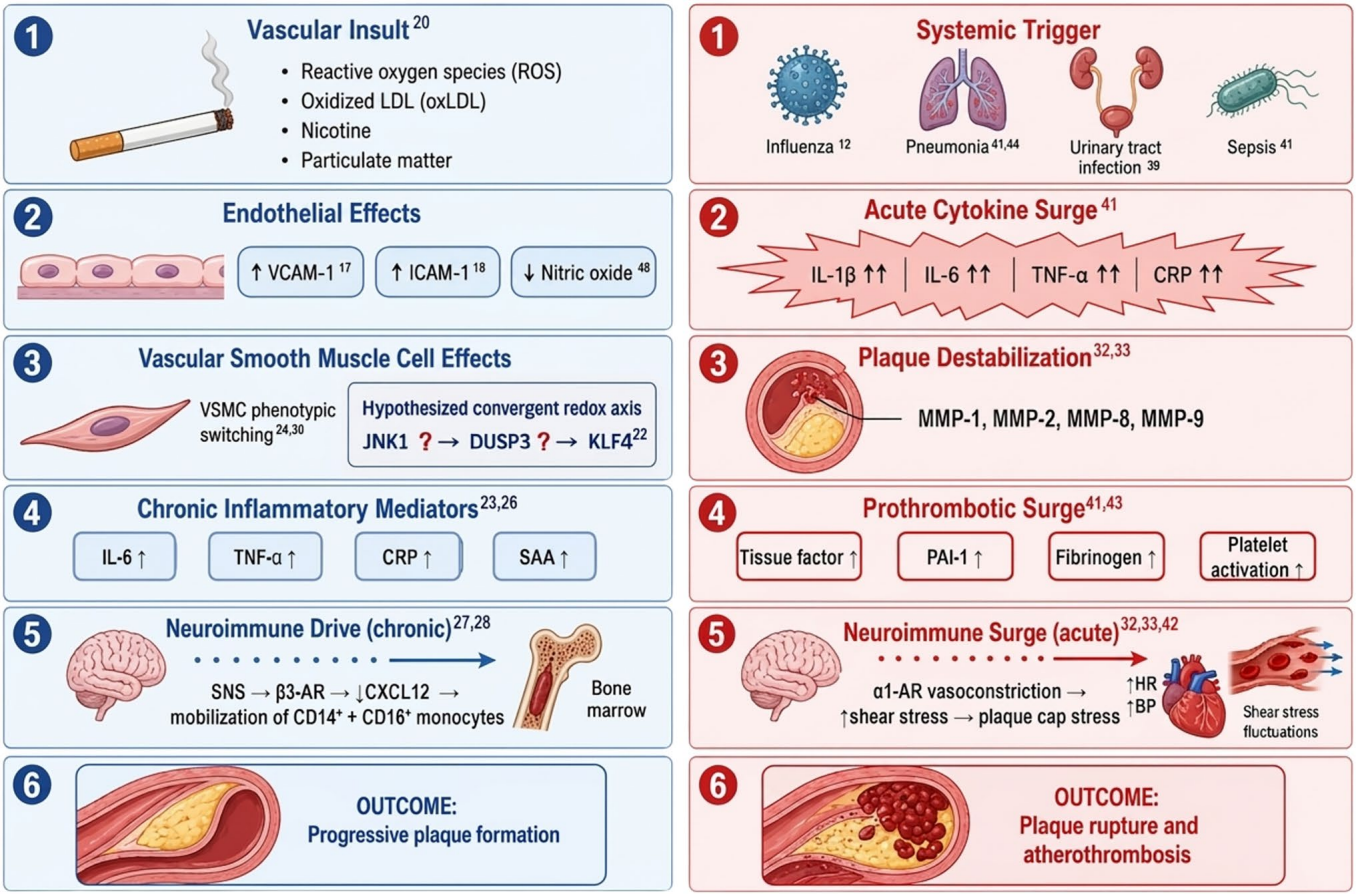
Dual inflammatory model of atherosclerosis: chronic vs. acute pathways. (A) Chronic Inflammation — Smoking-Induced Atherogenesis. Sustained tobacco-derived exposure [20] drives endothelial dysfunction (↑VCAM-1 [17], ↑ICAM-1 [18], ↓NO [48]) and VSMC phenotypic switching [24, 30]. A hypothesized convergent redox axis characterized in abdominal aortic aneurysm (JNK1 → DUSP3 → KLF4 [22]) is proposed as a candidate driver of VSMC switching in smoking-induced atherogenesis. Chronic elevation of IL-6, TNF-α, CRP, and SAA [23, 26] together with sympathetic-mediated mobilization of CD14⁺⁺CD16⁺ monocytes (β₃-AR → ↓CXCL12) [27, 28] promotes progressive plaque formation. (B) Acute Inflammation — Infection-Triggered Atherothrombosis. Acute infections (influenza [12], pneumonia [41, 44], UTI [39], sepsis [41]) trigger systemic cytokine surges [41], plaque destabilization with MMP release [32, 33], a prothrombotic surge (↑tissue factor, ↑PAI-1, ↑fibrinogen, ↑platelet activation) [41, 44], and a neuroimmune surge with α₁-AR-driven shear-stress fluctuations [32, 33, 42], culminating in fibrous cap rupture and atherothrombosis. Created in BioRender. Benchimol Barbosa, P. R. (2026) https://BioRender.com/tm3nqvo. α₁/β₃-AR, alpha-1/beta-1/beta-3 adrenergic receptor; CRP, C-reactive protein; CXCL12, C-X-C motif chemokine ligand 12; DUSP3, dual-specificity phosphatase 3; ICAM-1, intercellular adhesion molecule-1; IL, interleukin; JNK1, c-Jun N-terminal kinase 1; KLF4, Krüppel-like factor 4; MMP, matrix metalloproteinase; NO, nitric oxide; PAI-1, plasminogen activator inhibitor-1; SAA, serum amyloid A; SNS, sympathetic nervous system; TNF-α, tumor necrosis factor-alpha; UTI, urinary tract infection; VCAM-1, vascular cell adhesion molecule-1; VSMC, vascular smooth muscle cell

One of the earliest vascular effects of cigarette smoking is endothelial dysfunction, primarily driven by impaired nitric oxide (NO) bioavailability1 [14]. NO is an endothelial-derived vasodilator and anti-inflammatory molecule; its reduction leads to increased vasoconstriction, platelet aggregation, and leukocyte adhesion. Specific tobacco smoke constituents, particularly nicotine and reactive oxygen species (ROS), upregulate the expression of adhesion molecules, including vascular cell adhesion molecule-1 (VCAM-1), intercellular adhesion molecule-1 (ICAM-1), and E-selectin, on the endothelial surface [17, 18]. This facilitates the adhesion and transendothelial migration of monocytes into the intima, where they differentiate into macrophages [19]. These macrophages ingest modified lipoproteins, forming foam cells and setting the stage for the development of fatty streaks. Figure 1 schematically depicts chronic vascular inflammation associated with smoking and the acute systemic immune responses triggered by infection, emphasizing their distinct but converging roles in promoting atherogenesis and plaque destabilization.

### Oxidative Stress, LDL Oxidation, and Vascular Smooth Muscle Cell Phenotypic Switching

Tobacco smoke contains high levels of ROS, including superoxide anions and reactive aldehydes, which directly contribute to oxidative stress within the vascular wall [20]. ROS oxidize circulating low-density lipoproteins (LDL) in the subendothelial space, generating oxidized LDL (OxLDL), a key trigger of the atherogenic cascade. OxLDL is chemotactic for monocytes, induces endothelial expression of scavenger receptors, and stimulates the release of proinflammatory cytokines and matrix metalloproteinases (MMP) from activated macrophages [21]. Furthermore, ROS promote endothelial apoptosis and impair vascular smooth muscle cell repair.

Beyond LDL oxidation, vascular ROS exert direct effects on vascular smooth muscle cells (VSMCs), promoting phenotypic switching from a contractile to a synthetic, migratory state. Synthetic VSMCs migrate from the media to the intima, secrete extracellular matrix components, and contribute to neointima formation and progressive plaque growth, complementing the α1-adrenergic-driven VSMC migration described in Sect. "Local Drive: Adventitial Innervation and Direct Arterial Wall Effects". At the molecular level, a redox-sensitive signaling axis involving JNK1, DUSP3, and KLF4 has been proposed to govern this contractile-to-synthetic transition [22]. Using a chemogenetic mouse model in which endothelial hydrogen peroxide generation produces vascular oxidative stress, Das et al. demonstrated that DUSP3-mediated dephosphorylation of JNK1 permits KLF4 nuclear translocation and VSMC phenotypic switching, with pharmacological inhibition of DUSP3 completely blocking abdominal aortic aneurysm formation [22]. Although this axis was established in a model of aortic aneurysm formation, VSMC phenotypic plasticity is a shared mechanistic step in atherogenesis, and whether the same signaling node operates in smoking-induced atherosclerosis remains to be determined.

### Chronic Cytokine Elevation and Systemic Inflammation

Chronic exposure to tobacco smoke constituents, mainly particulate matter, ROS, and nicotine, drives sustained systemic inflammation. The circulating levels of interleukin-6 (IL-6), tumor necrosis factor-alpha (TNF-α), C-reactive protein (CRP), and serum amyloid A (SAA) are consistently elevated in smokers compared to non-smokers. These cytokines play a dual role: systemically, they maintain a proinflammatory milieu; locally, they promote smooth muscle cell proliferation, collagen degradation, and further recruitment of leukocytes to the vascular wall [23, 24]. IL-6 and CRP levels are correlated with increased cardiovascular risk and are considered biomarkers of vascular inflammation [25, 26].

### Sympathetic Activation: From Hematopoietic Drive to Arterial Remodeling

#### Systemic Drive: Bone Marrow Reprogramming and Monocyte Mobilization

**Fig. 2:**
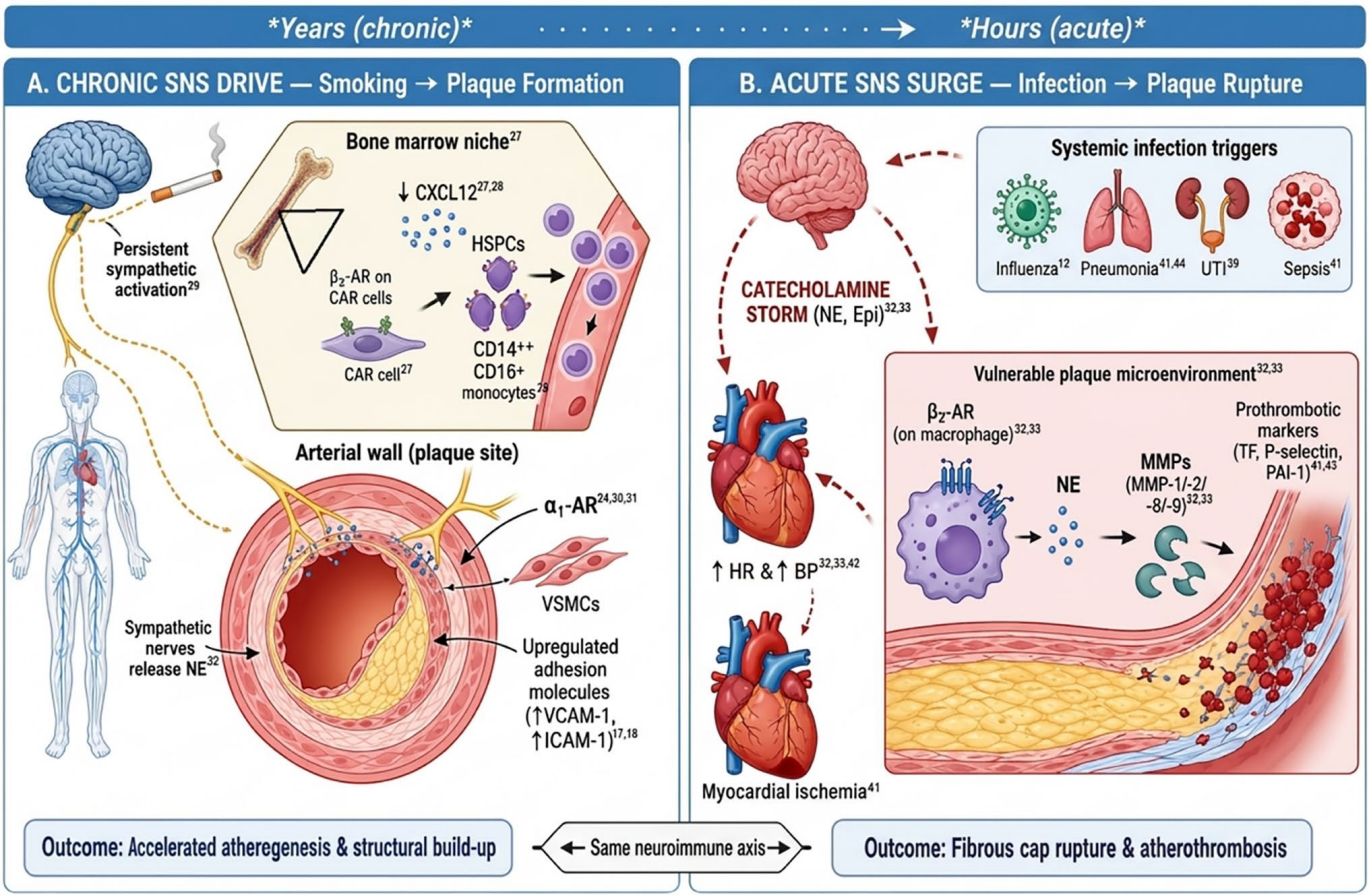
Dual role of sympathetic activation in atherosclerotic plaque dynamics. The same neuroimmune axis is activated in two patterns: chronic drive in smoking (A, years) versus acute surge in infection (B, hours). (A) Chronic SNS Drive. Persistent sympathetic activation [29] acts on bone marrow β₃-adrenergic receptors of CAR cells [27], suppressing CXCL12 [27, 28] and mobilizing CD14⁺⁺CD16⁺ monocytes [28]. At the arterial wall, adventitial sympathetic NE release [32] engages α₁-AR on VSMCs [24, 30, 31] and upregulates endothelial adhesion molecules (↑VCAM-1, ↑ICAM-1) [17, 18], yielding accelerated atherogenesis. (B) Acute SNS Surge. Systemic infections (influenza [12], pneumonia [41, 44], UTI [39], sepsis [41]) precipitate a catecholamine storm [32, 33] with ↑HR/↑BP [32, 33, 42]. Within the vulnerable plaque microenvironment [32, 33], NE binds β₂-AR on macrophages [32, 33], releasing MMPs (MMP-1, MMP-2, MMP-8, MMP-9) [32, 33] and triggering a prothrombotic surge (↑TF, ↑P-selectin, ↑PAI-1) [41, 44], culminating in myocardial ischemia [41], fibrous cap rupture, and atherothrombosis. α₁/β₂/β₃-AR, alpha-1/beta-2/beta-3 adrenergic receptor; BP, blood pressure; CAR, CXCL12-abundant reticular (cell); CXCL12, C-X-C motif chemokine ligand 12; HR, heart rate; ICAM-1, intercellular adhesion molecule-1; MMP, matrix metalloproteinase; NE, norepinephrine; PAI-1, plasminogen activator inhibitor-1; SNS, sympathetic nervous system; TF, tissue factor; UTI, urinary tract infection; VCAM-1, vascular cell adhesion molecule-1; VSMC, vascular smooth muscle cell. Created in BioRender. Benchimol Barbosa, P. R. (2026) https://BioRender.com/tm3nqvo

Long-term exposure to tobacco smoking is associated with long-lasting sympathetic nervous system (SNS) overactivity, which exerts a profound systemic drive on atherogenesis by reprogramming hematopoietic niches [27]. The bone marrow is densely innervated by sympathetic nerve fibers that terminate in close proximity to hematopoietic stem and progenitor cells (HSPCs) and their regulatory stromal niches. Under stress or chronic sympathetic stimulation, norepinephrine (NE) targets β3-adrenergic receptors expressed on CXCL12-abundant reticular (CAR) cells and osteoblasts. Activation of the β3-adrenergic receptor suppresses the synthesis of CXCL12 (SDF-1), a critical chemokine responsible for the retention of HSPCs within the marrow via the CXCR4 receptor [27] (Fig. 2). Downregulation of the CXCL12/CXCR4 axis leads to massive mobilization of HSPCs into the circulation and their subsequent homing to the spleen, where they undergo extramedullary hematopoiesis [28]. The splenic reservoir continuously provides the circulation with primed inflammatory monocytes, resulting in a sustained output of inflammatory leukocytes, particularly Ly6C-high monocytes (in mice), CD14^++^CD16^+^ monocytes (in humans), and neutrophils. These cells are primed for migration and exhibit increased expression of adhesion molecules, creating a “supply-side” pressure that accelerates plaque formation [29].

#### Local Drive: Adventitial Innervation and Direct Arterial Wall Effects

In addition to the systemic mobilization of immune cells, sympathetic stimulation exerts direct and local effects on arterial wall structure. The vascular adventitia is richly innervated by sympathetic efferent fibers that release neurotransmitters that diffuse into the media and intima (Fig. 2).

i. Vascular Smooth Muscle Cell (VSMC) Migration and Phenotypic Switching: Acting primarily through α1-adrenergic receptors, NE promotes phenotypic switching of VSMCs from a contractile (quiescent) state to a synthetic migratory phenotype. This stimulation enhances VSMC migration from the media to the intima, which is a key event in neointima formation and plaque remodeling [30, 31].
ii. Endothelial Activation: Sympathetic mediators induce endothelial dysfunction by promoting the upregulation of ICAM-1 and VCAM-1, enabling the attraction and diapedesis of an expanded pool of circulating monocytes [32].
iii. Adventitial Crosstalk: Recent evidence suggests that adventitial sympathetic nerves also communicate with resident immune cells (macrophages and T cells) in the outer artery wall, promoting the release of neurotrophins (such as NGF) and cytokines that perpetuate local inflammation and nerve sprouting, creating a vicious neuroinflammatory cycle [33].

### Emerging Soluble Inflammatory Mediators: Soluble Triggering Receptor Expressed on Myeloid Cells -Like Transcript-1 (sTLT1) and Peptidoglycan Recognition Protein 2 (PGLYRP2)

Recent proteomic profiling of patients with coronary artery disease and acute coronary syndromes has identified soluble inflammatory proteins that bridge platelet activation, innate immune sensing, and the chronic inflammatory milieu sustained by smoking. Two of these mediators, soluble triggering receptor expressed on myeloid cells-like transcript-1 (sTLT1) and peptidoglycan recognition protein 2 (PGLYRP2), deserve particular attention given their mechanistic relevance to the framework developed here [34, 35].

sTLT1 is released from activated platelet α-granules following ADAM17-mediated cleavage. Circulating sTLT1 binds to the FcγRI receptor on macrophages and activates the SYK–MEK/ERK–NF-κB signaling cascade, leading to sustained TNF-α secretion. In atherosclerotic apoE−/− mice, plasma sTLT1 and TNF-α concentrations progressively increase with lesion advancement, supporting the role of sTLT1 as a platelet-derived amplifier of macrophage inflammation in chronic vascular disease [34].

Although direct evidence of sTLT1 elevation in smokers is not yet available, the convergence of these pathways suggests that sTLT1 may represent a biologically plausible mechanistic link between smoking-induced platelet hyperreactivity and the perpetuation of macrophage-driven plaque inflammation [34]. Direct measurement of plasma sTLT1 levels in active smokers and longitudinal studies on smoking cessation are warranted to confirm this proposed connection.

PGLYRP2, a hepatic N-acetylmuramoyl-L-alanine amidase that hydrolyzes circulating bacterial peptidoglycan, is dysregulated in the plasma of patients with ST-segment elevation myocardial infarction, along with proteins involved in reverse cholesterol transport [35]. This pattern positions PGLYRP2 at the interface between innate immune sensing of microbial-derived molecular patterns and lipid homeostasis, providing a candidate molecular link between low-grade infectious exposure, frequently coexistent in smokers, and impaired cholesterol efflux that underlies foam cell formation. This innate immune connection also establishes a conceptual bridge with the direct pathogen-driven mechanisms discussed in Sect. "Pathogen-Driven Atherogenesis and Smoking-Induced Microbial Dysbiosis".

### Quantitative Impact and Population-Level Consequences

Epidemiological data consistently demonstrate that smoking increases the risk of coronary artery disease and myocardial infarction by 2–4 times compared with non-smokers^35^. According to the Global Burden of Disease 2021 report, cigarette smoking accounts for approximately 20% of all atherosclerotic cardiovascular deaths globally [36, 37]. These figures highlight the global scale and enduring impact of smoking-induced vascular injury.

## Infection-Associated Inflammation and Acute Atherothrombosis

The association between acute infections and cardiovascular events has gained increasing attention in the past three decades. Historically, infections were viewed as unrelated to coronary artery disease and presumed to affect distinct physiological systems. However, large-scale epidemiological studies in the early 2000 s, such as those by Kwong et al. [12] and Katsoularis et al. [38], demonstrated a significantly increased risk of myocardial infarction and stroke following respiratory or urinary tract infections [39, 40]. These findings position acute systemic inflammation as a transient yet potent amplifier of cardiovascular risk.

Mechanistically, the inflammatory cascade induced by infection differs fundamentally from that induced by smoking. While cigarette smoke chronically targets the endothelium and vascular wall with localized oxidative stress and immune activation, infections elicit acute and systemic inflammatory responses. These can trigger endothelial dysfunction and thrombotic cascades that destabilize vulnerable atherosclerotic plaques, rather than initiating new ones.

### Systemic Cytokine Bursts

Acute infections induce rapid and robust increases in circulating cytokines, notably IL-6, interleukin-1β (IL-1β), TNF-α, and acute-phase reactants, such as CRP. CRP levels may exceed 31 mg/L within the first 48–72 h of infection [41]. These cytokines promote endothelial cell activation, increase vascular permeability, and express adhesion molecules, facilitating monocyte adhesion and infiltration, even in areas with previously subclinical atherosclerosis.

### Plaque Vulnerability and Matrix Degradation

Systemic inflammation potentiates local responses to vascular lesions. Cytokines such as IL-1 and TNF-α upregulate MMPs, particularly MMP-1, MMP-2, MMP-8, and MMP-9, in the fibrous cap. These proteolytic enzymes degrade structural collagen, weakening the plaque architecture and increasing the likelihood of rupture [42] (Fig. 2, panel B). Acute infection and systemic inflammation drive increased macrophage activation and tissue factor expression within atherosclerotic plaques, generating a prothrombotic microenvironment [41, 43].

### Sympathetic Surge and Plaque Destabilization

Along with the release of prothrombotic mediators, acute sympathetic “storm” triggered by severe infection also acts as a potent destabilizer of the hemostatic balance [29, 41, 42]. Pathophysiologically, fever and systemic inflammatory responses associated with infections, such as influenza or pneumonia, drive a surge in stress hormones. This results in a hemodynamic mismatch, where tachycardia and hypertensive surges increase wall shear stress, physically straining the fibrous cap of atheromas.

However, a more insidious molecular mechanism operates simultaneously with this process. Macrophages residing in atherosclerotic plaques express β2-adrenergic receptors. When stimulated by a surge in stress hormones during infection, these macrophages release high concentrations of MMPs and proinflammatory cytokines. This neuroimmune signaling accelerates the degradation of the collagen matrix that stabilizes the fibrous cap, effectively converting a stable lesion into a vulnerable one precisely when hemodynamic stress is the highest [32, 33, 42] (Fig. 2).

Parallel to this neuroimmune destabilization, systemic inflammation during acute infections increases the levels of circulating prothrombotic mediators, including fibrinogen, D-dimer, von Willebrand factor, and platelet activation markers. The net effect is heightened blood coagulability and an increased risk of intravascular thrombosis upon plaque rupture [41].

### Epidemiologic Risk Evidence

Multiple population-based studies have supported the temporal relationship between infection and acute coronary events. For example, a case-crossover study by Kwong et al.^12^ demonstrated that the risk of acute myocardial infarction was six times higher in the first week following laboratory-confirmed influenza infection. Similarly, Katsoularis et al. [38] reported a significant increase in myocardial infarction and stroke in the two weeks following acute respiratory or urinary infections [39]. Earlier observational work by Musher et al. [44] further documented a high incidence of acute cardiac events, including myocardial infarction, arrhythmia, and worsening heart failure, in patients hospitalized for community-acquired pneumococcal pneumonia, establishing one of the earliest empirical links between bacterial respiratory infection and acute cardiac morbidity. These associations consistently show a peak in cardiovascular events shortly after infection, with the risk returning to baseline within 2–4 weeks [44].

### Long-Term Implications of Recurrent and Low-Grade Infections

Although the short-term cardiovascular risks posed by acute infections are well established, the long-term implications of recurrent or low-grade infections remain unclear [12, 41]. It remains unclear whether conditions such as asymptomatic bacteriuria, frequent upper respiratory infections, and mild periodontal disease contribute to sustained vascular inflammation and atherogenesis. Moreover, infection-driven hematopoietic shifts, such as trained immunity or monocytosis, may influence plaque progression and repair, warranting further prospective and mechanistic studies.

Beyond cytokine and adrenergic responses, host innate immunity involves pattern-recognition systems that monitor circulating microbial-derived molecules. PGLYRP2 hydrolyzes plasma peptidoglycan and is therefore particularly relevant in low-grade infectious states, where continuous translocation of pathogen-associated molecular patterns into the bloodstream sustains this enzymatic surveillance [35] (Sect. "Emerging Soluble Inflammatory Mediators: Soluble Triggering Receptor Expressed on Myeloid Cells -Like Transcript-1 (sTLT1) and Peptidoglycan Recognition Protein 2 (PGLYRP2)"). Innate microbial sensing thus emerges as a third mechanistic axis, alongside cytokines and neuroimmune signaling, through which recurrent infectious exposure may modulate atherosclerotic risk (see also Sect. "Pathogen-Driven Atherogenesis and Smoking-Induced Microbial Dysbiosis").

Table 1 summarizes the inflammatory pathways in smoking- and infection-induced atherosclerosis.

**Table 1:** Comparative summary of inflammatory pathways in smoking- and infection-induced atherosclerosis

Characteristic	Smoking-induced inflammation	Infection-associated inflammation
Temporal profile	Chronic (long-term exposure) [20]	Acute (short-term spikes) [12]
Trigger type	Tobacco smoke and its chemical constituents [20]	Bacterial or viral pathogens [41]
Primary site of action	Endothelial and vascular wall [17, 19, 45]	Systemic (multiple tissues and organs) [38, 41]
Key inflammatory mediators	IL-6, TNF-α, CRP, SAA, ROS, oxLDL [21, 23, 26]	IL-6, IL-1β, TNF-α, CRP, MMPs [38, 41]
Effect on endothelium	Direct injury and persistent dysfunction via ROS and NO suppression [17, 19, 45, 48]	Transient activation and permeability increase via cytokine surge [38, 41]
Immune cell recruitment	Monocytes → foam cells; M1 macrophages [17, 20, 46]	Monocytes, neutrophils; systemic activation [38, 41]
Matrix remodeling	Progressive MMP activity with plaque growth [47]	Acute MMP surge destabilizing plaques [42]
Prothrombotic effects	Platelet activation, fibrin deposition (chronic) [48]	Acute coagulation surge: D-dimer, fibrinogen [38, 41]
Sympathetic nervous system role	Chronic Drive: Mobilizes stem cells from bone marrow and promotes smooth muscle migration (neointima formation) [27, 30, 31]	Acute Surge: Increases hemodynamic shear stress and triggers macrophage-mediated collagen degradation (plaque rupture) [32, 41, 42]
Atherogenesis role	Initiates and sustains plaque formation [17, 19]	Triggers rupture of pre-existing plaques [38, 41]
Epidemiologic impact	~ 2–4× risk increase; ~20% of global ASCVD deaths [36, 37]	5–6× MI risk increase post-infection (short-term) [12, 13, 44]

IL-6 – interleukin 6, IL-1β – interleukin 1β, TNF-α – tumor necrosis factor alpha, CRP – C-reactive protein, ROS – reactive oxygen species, oxLDL – oxidized low density lipoprotein, NO – nitric oxide, MMP – matrix metalloproteinases, ASCVD - atherosclerotic cardiovascular disease, MI – myocardial infarction

## Pathogen-Driven Atherogenesis and Smoking-Induced Microbial Dysbiosis

Previous studies have explored whether certain bacterial pathogens directly contribute to atherogenesis by colonizing vascular tissues. Organisms such as Chlamydia pneumoniae and Helicobacter pylori have been detected in atherosclerotic plaques, and experimental models suggest they can infect endothelial or smooth muscle cells, promote foam cell formation, and trigger inflammatory cytokine production [49–52]. These findings generated enthusiasm for a pathogen-driven model of atherosclerosis; however, the absence of consistent causal evidence, unclear mechanisms of vascular localization, and limited clinical translation tempered this hypothesis. Notably, several large-scale randomized controlled trials, including WIZARD, ACES, and PROVE IT–TIMI 22 gatifloxacin arm, failed to demonstrate cardiovascular benefits from antibiotic therapy targeting Chlamydia pneumoniae in patients with coronary artery disease, effectively closing the door on direct antimicrobial approaches to atherosclerosis prevention [53]. Nevertheless, these studies broadened the inflammatory paradigm of atherogenesis beyond traditional risk factors and continue to inspire interest in non-traditional proinflammatory stimuli.

While early work focused on a small number of specific pathogens, broader microbial profiling of human atherosclerotic lesions has progressively expanded the inventory of bacteria associated with plaque tissue. A pivotal meta-analysis pooling 63 studies and 1,791 patients confirmed the presence of 23 oral commensal bacteria within coronary and carotid atherosclerotic plaques, with *Campylobacter rectus*, *Porphyromonas gingivalis*, *Porphyromonas endodontalis*, *Prevotella intermedia*, and *Prevotella nigrescens* uniquely associated with coronary plaques [54]. The recurrent detection of oral-derived organisms in vascular tissue has, in turn, raised the question of which factors shape the composition of the oral microbiome in the first place. Among modifiable exposures, chronic smoking has emerged as a major modulator: recent salivary metagenomic analysis demonstrated that smokers exhibit a distinct oral microbial signature, with enrichment of *Streptococcus*, *Prevotella*, *Veillonella*, *TM7x*, and *Porphyromonas* at the genus level compared with non-smokers [55]. The overlap between bacteria enriched in the oral cavity of smokers and bacteria recurrently detected in atherosclerotic plaques, notably *Porphyromonas* and *Prevotella* species, is consistent with population-level evidence linking the relative abundance of *Prevotella*, *Megasphaera*, and *Veillonella* to carotid intima-media thickness, with smoking identified as one of the principal modulators of oral microbial composition in the same cohort [56]. This convergence supports the hypothesis that smoking-induced microbial dysbiosis may represent a previously underrecognized contributor to atherogenesis. Whether this pathway acts as a causal driver or a parallel marker of cardiovascular risk in tobacco users remains uncertain, however, and the magnitude of any incremental risk has not been quantified. Comprehensive longitudinal studies integrating microbiome profiling, vascular imaging, and prospective cardiovascular outcomes are needed to clarify this mechanistically plausible yet underexplored axis to atherogenesis in tobacco users.

## Integrative Discussion

The preceding sections detail how chronic smoking and acute infections promote atherosclerosis through distinct inflammatory mechanisms. While these pathways differ in temporal dynamics and primary vascular targets, they converge at several critical neuroimmune nodes that merit explicit integration in future studies.

The sympathetic nervous system emerges as a central effector in both contexts, albeit through different mechanisms of action. Chronic sympathetic activation associated with long-term smoking targets the bone marrow niche, suppressing CXCL12 expression and mobilizing proinflammatory monocytes into circulation. Bone marrow-driven mobilization continuously fuels plaque growth through persistent leukocyte recruitment. In contrast, the acute sympathetic surge triggered by systemic infection exerts its effects locally within the arterial wall, where catecholamines directly activate β2-adrenergic receptors on plaque-resident macrophages, prompting MMP release and collagen degradation at the time of maximal hemodynamic stress. Thus, the same neuroimmune axis that builds plaques over years can rupture them within hours under different activation patterns [32, 33] (Fig. 2A and B).

Monocyte-macrophage biology represents a second point of convergence. Smoking skews hematopoiesis toward inflammatory monocyte subsets (CD14^++^CD16^+^ from Sect. "Systemic Drive: Bone Marrow Reprogramming and Monocyte Mobilization") that exhibit increased migratory and phagocytic capacities. Infection-induced cytokine surges further activate these cells within the plaques, amplifying the release of MMP implicated in acute plaque destabilization (Sect. "Emerging Soluble Inflammatory Mediators: Soluble Triggering Receptor Expressed on Myeloid Cells -Like Transcript-1 (sTLT1) and Peptidoglycan Recognition Protein 2 (PGLYRP2)" and "Sympathetic Surge and Plaque Destabilization"). In individuals who smoke and experience recurrent infections, this convergence may create a particularly adverse milieu with a larger pool of primed inflammatory cells encountering repeated activation signals.

Endothelial dysfunction constitutes the third shared pathway [14, 21]. Chronic smoking impairs nitric oxide bioavailability, upregulating adhesion molecules and facilitating continuous monocyte diapedesis [17, 18, 20, 44, 47]. Acute infections superimpose transient but intense endothelial activation through circulating cytokines, further increasing vascular permeability and leukocyte adhesion at the sites of preexisting injury [24, 25, 40, 41].

From a clinical perspective, this integrated neuroimmune framework suggests that cardiovascular risk is best understood by examining combined exposures. The chronic inflammatory burden imposed by smoking establishes a vulnerable substrate, whereas episodic infectious insults provide a destabilizing trigger [23, 26, 41, 42, 57]. Recognizing this interplay has therapeutic implications, and interventions targeting sympathetic tone, monocyte trafficking, or residual inflammatory risk [57] are necessary to address both chronic and episodic components of atherogenesis to achieve optimal cardiovascular protection.

While this integrated framework provides a coherent mechanistic narrative, several elements, particularly those involving emerging mediators and low-grade infectious exposures, remain incompletely demonstrated and are further discussed in the Limitations section.

## Limitations

This narrative review aimed to describe the mechanistic evidence of chronic atherosclerotic plaque development promoted by smoking and plaque destabilization triggered by acute inflammatory surges. Study selection was guided by thematic relevance rather than by systematic screening protocols, with the aim of providing a descriptive mechanistic framework.

Among the conceptual limitations of this synthesis, the proposed mechanistic role of sTLT1 (Sect. "Emerging Soluble Inflammatory Mediators: Soluble Triggering Receptor Expressed on Myeloid Cells -Like Transcript-1 (sTLT1) and Peptidoglycan Recognition Protein 2 (PGLYRP2)) in smoking-related atherogenesis, the integration of PGLYRP2-mediated innate microbial sensing within low-grade infectious states [34, 35, 48, 50, 51] (Sect. "Long-Term Implications of Recurrent and Low-Grade Infections"), and the extrapolation of the JNK1–DUSP3–KLF4 redox axis from abdominal aortic aneurysm models to smoking-induced VSMC switching [22] (Fig. 1) remain biologically plausible but empirically untested. These hypotheses warrant prospective experimental and clinical validation, particularly through targeted plasma biomarker studies in smokers and longitudinal cohorts of recurrent infectious exposure.

This methodological approach prioritized coherent conceptual integration rather than exhaustive coverage to guide and refine the information structure and evidence-based knowledge. While this allowed for an in-depth exploration of neuroimmune pathways, it also meant that other mechanistic pathways and alternative interpretations may have received less emphasis.

## Conclusion and Future Directions

The interplay between inflammation and atherosclerosis is multifaceted, and the present review highlights how chronic (smoking) and acute (infection) inflammatory pathways, side by side, show how each shapes plaque biology in a distinct way. Cigarette smoking represents a sustained inflammatory insult that initiates and propagates atherogenesis through endothelial injury, oxidative stress, immune cell recruitment, sustained sympathetic activation, and hematopoietic reprogramming. In contrast, systemic infections and other acute inflammatory states act as transient amplifiers of cardiovascular risk by promoting sympathetic surge that destabilizes vulnerable plaques, enhances thrombosis, and precipitates acute coronary syndromes. These inflammatory triggers differ not only in temporal dynamics (chronic versus episodic) but also in their molecular targets and vascular consequences [15, 42]. This neuroimmune framework extends conventional inflammatory models by identifying the sympathetic nervous system as a shared effector driving both plaque formation and destabilization.

A particularly promising avenue for future investigation concerns the JNK1-DUSP3-KLF4 signaling axis. Direct validation in atherosclerotic models, together with assessment of DUSP3 as a potential therapeutic target, is a promising line of investigation that is worth exploring in preclinical studies before clinical translation is considered [22].

Smoking represents a major modifiable driver of chronic vascular inflammation, whereas acute infection acts as an episodic destabilizing counterpart; converging on neuroimmune activation as a shared mediating mechanism, both constitute central targets for cardiovascular prevention and treatment.

## Data Availability

Not applicable. No original data were generated or analyzed in this structured narrative review.
